# Integrated Metabolomic and Transcriptomic Analyses Reveal Novel Insights of Anthocyanin Biosynthesis on Color Formation in Cassava Tuberous Roots

**DOI:** 10.3389/fnut.2022.842693

**Published:** 2022-04-05

**Authors:** Lili Fu, Zehong Ding, Weiwei Tie, Jinghao Yang, Yan Yan, Wei Hu

**Affiliations:** ^1^Hainan Key Laboratory for Biosafety Monitoring and Molecular Breeding in Off-Season Reproduction Regions, Key Laboratory of Biology and Genetic Resources of Tropical Crops, Institute of Tropical Bioscience and Biotechnology, Chinese Academy of Tropical Agricultural Sciences, Haikou, China; ^2^Hainan Key Laboratory for Protection and Utilization of Tropical Bioresources, Hainan Institute for Tropical Agricultural Resources, Chinese Academy of Tropical Agricultural Sciences, Haikou, China; ^3^Sanya Research Institute of Chinese Academy of Tropical Agricultural Sciences, Sanya, China

**Keywords:** cassava tuberous roots, color formation, anthocyanin biosynthesis, metabolome, transcriptome, coordinate regulation

## Abstract

Yellow roots are of higher nutritional quality and better appearance than white roots in cassava, a crucial tropical and subtropical root crop. In this work, two varieties with yellow and white cassava roots were selected to explore the mechanisms of color formation by using comparative metabolome and transcriptome analyses during seven developmental stages. Compared with the white-rooted cassava, anthocyanins, catechin derivatives, coumarin derivatives, and phenolic acids accumulated at higher levels in yellow-rooted cassava. Anthocyanins were particularly enriched and displayed different accumulation patterns during tuberous root development. This was confirmed by metabolic comparisons between five yellow-rooted and five white-rooted cassava accessions. The integrative metabolomic and transcriptomic analysis further revealed a coordinate regulation of 16 metabolites and 11 co-expression genes participating in anthocyanin biosynthesis, suggesting a vital role of anthocyanin biosynthesis in yellow pigmentation in cassava tuberous roots. In addition, two transcriptional factors, i.e., *MeMYB5* and *MeMYB42*, were also identified to co-express with these anthocyanin biosynthesis genes. These findings expand our knowledge on the role of anthocyanin biosynthesis in cassava root color formation, and offer useful information for the genetic breeding of yellow-rooted cassava in the future.

## Introduction

Cassava (*Manihot esculenta*) is one of the highly used tropical and subtropical root crops, providing staple food for more than 800 million people worldwide (1). Cassava tuberous roots are high in starch but very low in protein, micronutrients, and bioactive compounds such as carotenoids and anthocyanins (2, 3). Cassava is typically white rooted, although a few yellow landraces have been reported in Amazonia, Brazil (4). Compared with white cassava roots, yellow cassava roots usually have higher levels of carotenoid contents (2, 5), flavanones, anthocyanins, and proanthocyanidins (6). In addition to the higher nutritional values, yellow cassava roots are also gaining popularity among consumers for their striking color compared to the white cassava roots (7). To date, the molecular mechanisms of color formation in cassava tuberous roots remain elusive, which greatly limits its breeding for higher nutritional contents.

Many studies have been conducted to investigate the color formation of yellow cassava roots. For instance, Carvalho et al. (5) characterized carotenoid profiles in 23 landraces of cassava tuberous roots with white-to-yellow-to-pink color. They established potential links of low transcript abundance of *LCYb* and *HYb* to the pink and yellow landraces, respectively. Similarly, Olayide et al. (8) found that carotenoid biosynthesis genes were expressed in both yellow and white cassava roots. Still, only lycopene-ε-cyclase (*LCY*ε), phytoene synthase 2 (*PSY2*), and β-carotenoid hydroxylase (*CHY*β) showed higher expression in yellow roots. Welsch et al. (2) revealed that a single nucleotide polymorphism in *PSY2* resulted in the accumulation of provitamin A carotenoids and induced yellow color to the cassava roots. Beyene et al. (9) enhanced β-carotene contents in cassava tuberous roots by co-expression of transgenes for deoxy-D-xylulose-5-phosphate synthase (*DXS*) and bacterial phytoene synthase (*crtB*). This resulted in color change of the cassava tuberous roots from white to yellow. These studies mainly focused on the carotenoid pathways; however, the carotenoid contents were not always higher in yellow cassava roots than white cassava roots (5), indicating that other pigments may also influence the coloring of yellow cassava roots.

Anthocyanins are well-known water-soluble phenolic pigments that color the fruits and flowers of many plants (10, 11). These pigments are present in vacuoles, and their hue and stability are usually influenced by intravacuolar environments, including pH, co-pigmentation, and complex formation with metal ions (11). In addition to the red, blue, and purple color formation, anthocyanins are reported to participate in color formation of yellow flowers in Herbaceous peony (12) and yellow peel in several fruits (13, 14). Similarly, in cassava, the anthocyanins and proanthocyanidins were more accumulated in yellow tuberous roots than white tuberous roots. Moreover, the expression of several anthocyanin biosynthesis genes, including *CHI, F3*′*5*′*H*, *F3H*, and *DFR*, was up-regulated in yellow cassava roots than white cassava roots (6). These results suggested that anthocyanins might also participate in the color formation of yellow cassava roots; however, the underlying key genes and regulatory networks remain unknown.

Due to the advantage of multi-omics in explaining complex biological problems, the integrated metabolomic and transcriptomic analyses have been widely applied to identify crucial genes and pathways controlling pigment accumulation in plants. For example, combined metabolome and transcriptome analyses were performed in pepper and asparagus cultivars, respectively, to demonstrate the roles of carotenoid and anthocyanin biosynthesis genes in color formation (15, 16). Similarly, the mechanisms underlying peel and pulp color formation were unveiled in pitaya fruit by an integrated transcriptome and metabolome approach, providing several candidate genes and metabolites for further functional characterization (13). In addition, network analysis of the metabolomic and transcriptomic profiles has been demonstrated as a powerful approach to uncover novel genes and regulatory pathways in potato pigmentation (17). However, no multi-omics studies have been conducted to determine the color formation in cassava tuberous roots, although there have been reports on drought response, root development, and nutritional properties (6, 18, 19).

In this work, comparative transcriptome and metabolome analyses were performed in seven developmental stages of SC205 (white-rooted cassava) and SC9 (yellow-rooted cassava), respectively, to explore the mechanisms of color formation in cassava tuberous roots. The findings will provide novel insights of anthocyanin biosynthesis on color formation in cassava tuberous roots and offer useful information for the genetic breeding of yellow-rooted cassava.

## Materials and Methods

### Plant Materials and Sample Collection

Two cassava varieties, i.e., SC9 and SC205, which have yellow and white tuberous roots, respectively, were used in this study. Cassava stems were sectioned with a length of about 15 cm each and planted in the Chinese Academy of Tropical Agricultural Sciences experimental farm under normal field conditions at Danzhou, China. As described previously (18), seven experimental blocks were designed. Each block consisted of four rows, and each row was planted with seven individual plants, which were regarded as different biological replicates. The typical roots of five plants cultivated in the middle of each row were sampled at ∼9 am, respectively, at a total of seven developmental stages (S1-S7) including 100, 140, 180, 220, 260, 300, and 340 days after planting. These time points roughly represented three critical stages of early (S1-S3), middle (S4), and late (S5-S7) during cassava production (20). Only one main root was collected for each plant. Each root was dissected into pieces of ∼3 mm thick from the middle, and then 5–6 pieces were immediately frozen in liquid nitrogen and stored at −80°C until analyzed.

### Metabolome Analysis

The untargeted metabolic experiments were performed at the Wuhan Metware Biotechnology Co., Ltd., as previously described (18, 21). Briefly, 100 mg powder was weighed and added to 1.2 ml 70% aqueous methanol for overnight extraction at 4°C. After centrifugation for 10 min at 10,000 *g*, the extracted solution was absorbed and filtrated. The quality control samples were prepared by mixing equal volumes of sample extracts and analyzed every 10 samples to monitor the repeatability. An ultra-performance liquid chromatography (UPLC) system (Shim-pack UFLC SHIMADZU CBM30A) and an MS/MS system (Applied Biosystems 6500 Q TRAP) were used to analyze the sample extracts under following conditions: UPLC column, Waters ACQUITY UPLC HSS T3 C18 (1.8 μm, 0.21 cm × 10 cm). The mobile phase consisted of pure water with 0.04% acetic acid as eluent A and acetonitrile with 0.04% acetic acid as eluent B. Sample measurement was executed with a gradient program employing the initial conditions of 95% A and 5% B. A linear gradient to 5% A and 95% B was programmed within 10 min, and the composition of 5% A and 95% B was maintained for 1 min. Subsequently, a composition of 95% A and 5% B was adjusted within 0.1 min and maintained for 2.9 min. The injection volume was fixed to 2 μL, and the column temperature was controlled at 40°C. The effluents were alternatively connected to electrospray ionization (ESI)-triple quadrupole-linear ion trap (Q TRAP)-MS.

The acquisitions of linear ion trap (LIT) and triple quadrupole (QQQ) scans were executed on a triple quadrupole-linear ion trap MS API 6500 Q TRAP LC/MS/MS system, equipped with a Turbo Ion-Spray interface (operating in positive ion mode and negative ion mode) and controlled by Analyst 1.6.3 software (AB Sciex). The ESI source operation parameters were following: ion source, turbo spray; source temperature, 550°C; ion spray voltage, 5,500 V for positive ion mode and −4,500 V for negative ion mode; ion source gas I, gas II, and curtain gas were set at 50, 60, and 30 psi, respectively; the collision gas was high. Instrument tuning and mass calibration were analyzed in QQQ and LIT modes using 10 and 100 μmol/L polypropylene glycol solutions, respectively. The acquisition of QQQ scan was executed during MRM experiment with collision gas (nitrogen) at 5 psi.

Metabolites were identified and relative quantified by searching the self-built MetWare database^1^ constructed based on the standard materials and purified compounds and the public databases (including MassBank, KNApSAcK, HMDB, MoTo DB, and METLIN), based on the accurate precursor ion (Q1) and production (Q3) values, m/z and MSMS spectra, retention time, and fragmentation pattern. The peak area of each chromatographic peak represented the relative content of the corresponding metabolite. All identified metabolites were used to conduct principle component analysis and orthogonal partial least squares discriminate analysis, and differentially accumulated metabolites (DAMs) were identified by setting the variable importance in projection (VIP) ≥ 1 and | log2(fold-change)| ≥ 1. Each sample was performed with three biological replications.

### Transcriptome Analysis

Library construction and RNA-seq sequencing were executed at the Annoroad Gene Technology Corporation (Beijing, China). In brief, total RNA was isolated from each sample to construct transcriptome libraries using Illumina TruSeq RNA sample prep Kit (Illumina, San Diego), according to the manufacturer’s instructions. RNA-seq libraries were sequenced on an Illumina Hiseq 4000 platform to generate 150 bp pair-end reads. Each sample was performed with three biological replicates.

As previously described (22, 23), sequence quality was checked by FastQC software^2^. Sequencing adaptors and low-quality bases were filtered by FASTX-toolkit^3^. Clean reads were mapped to the cassava reference genome (version 6.1) by HISAT2 v2.1.0 (24) with default parameters and then assembled by Stringtie v1.3.4 (24) with reference genome-based strategy. Differentially expressed genes (DEGs) were identified by DESeq2 (25) setting false discovery rate < 0.05 and log2 | fold-change| > 1. Gene expression was measured in fragments per kilobase per million mapped reads (FPKM).

### Metabolomic and Transcriptomic Integrative Analysis

The levels of metabolites and genes were log2-transformed for metabolomic and transcriptomic integrative analysis. The abundance patterns of metabolites and genes were determined by the standard procedure of WGCNA (26) based on the Pearson correlation coefficient and then visualized by R package “pheatmap.” Cassava genes were classified into distinct hierarchical categories using the MapMan annotation system (27) for their biological function interpretation. The significantly enriched categories were identified by Fisher’s exact test as previously reported (22, 23). WGCNA was also applied to identify the association between genes and metabolites, while Cytoscape software (28) was used for network visualization.

### qRT-PCR Analysis

The qRT-PCR experiments were executed as previously described (19) to verify the expression of RNA-seq. Total RNA was isolated using RNAiso reagent (OMEGA), and then reversely transcribed to obtain cDNA using PrimeScript RT reagent Kit with gDNA Eraser (Takara, Dalian, China). Eleven genes related to anthocyanin biosynthesis were selected and analyzed by qRT-PCR. The primers were listed in Supplementary Table 1.

The qRT-PCR was executed on a Stratagene Mx3000P machine (Stratagene, CA, United States) using SYBR Premix Ex Taq (Takara, Dalian, China) with the following processes: 30 s at 95°C, then 40 cycles of 10 s at 95°C and 30 s at 60°C. A thermal denaturing step was executed to produce the melt curves for verification of amplification specificity. The cassava actin gene was used as an internal control (19). Each sample was measured in triplicates, and the relative gene expression was calculated by the 2^–ΔΔCt^ method (23).

## Results

### Metabolomic Profiling of Cassava Tuberous Roots With Different Colors Across the Developmental Stages

**FIGURE 1:**
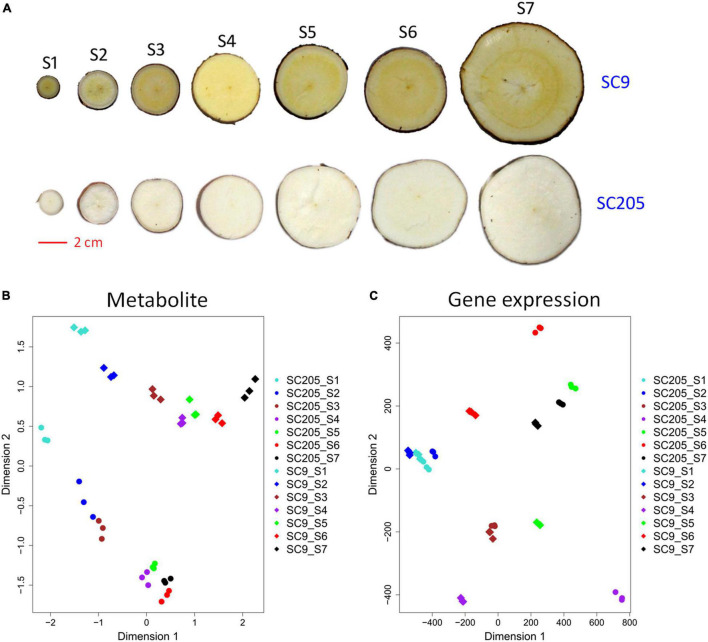
Overview of tuberous roots of SC9 and SC205. **(A)** Phenotypes of SC9 and SC205 tuberous roots collected at seven developmental stages from 100–340 days after planting: S1, 100; S2, 140; S3, 180; S4, 220; S5, 260; S6, 300; and S7, 340. The scale bar equals 2 cm. **(B,C)** Clustering of tuberous root samples based on the metabolite levels and gene expression profiles, respectively. The symbols with the same color represent different replicates of a sample.

In total, 488 metabolic compounds were identified and quantified in tuberous roots of SC9 and SC205 across seven developmental stages (S1-S7, Figure 1A). These metabolites were divided into fifteen categories with the three most abundant were flavones (108), amino acid derivatives (60), and lipids (56). Principle component analysis revealed that tuberous root samples of SC9 and SC205 were separated while different replicates were closely grouped (Figure 1B), indicating high reliability of our metabolomic data and a significant impact of metabolites on the color of tuberous roots.

**FIGURE 2:**
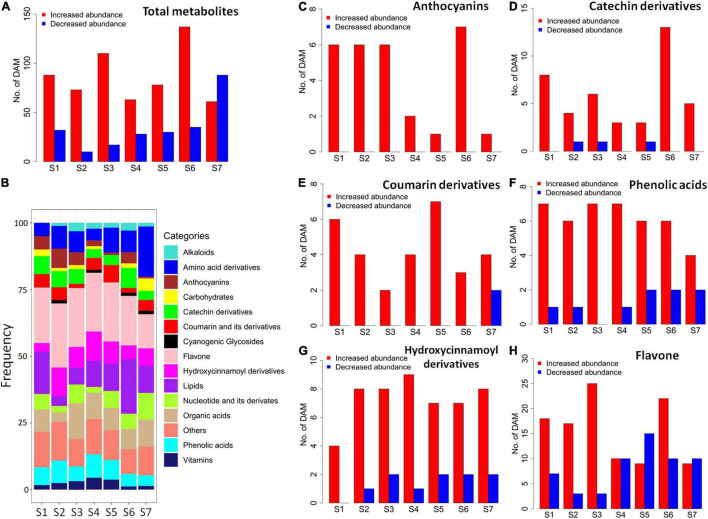
Differentially accumulated metabolites between SC9 and SC205 during tuberous root development. **(A)** Number of all abundance-increased and abundance-decreased DAMs, which showed higher and lower metabolite levels in SC9 than SC205, respectively. **(B)** Category frequency of all DAMs. **(C–H)** Number of abundance-increased and abundance-decreased DAMs relevant to anthocyanins, catechin derivatives, coumarin derivatives, phenolic acids, hydroxycinnamoyl derivatives, and flavones, respectively.

A total of 335 differentially accumulated metabolites (DAMs) were identified by metabolomic comparisons between SC9 and SC205 at seven developmental stages, respectively (Supplementary Table 2). Most metabolites were higher accumulated in SC9 compared with SC205 from S1 to S6, while this trend was reversed at S7 (Figure 2A). On average, the categories with most DAMs were flavones (22.84%) and lipids (12.27%), followed by amino acid derivatives (8.69%), organic acids (8.10%), hydroxycinnamoyl derivatives (7.2%), phenolic acids (6.32%), nucleotide and its derivatives (5.23%), catechin derivatives (4.79%), coumarin and its derivatives (3.60%), and anthocyanins (3.57%). However, their frequencies varied slightly across different developmental stages (Figure 2B).

The metabolomic changes in each category were also observed. Notably, anthocyanin-related DAMs were increased in SC9 than SC205 across all the developmental stages (Figure 2C). Likely, most DAMs related to catechin derivatives, coumarin and its derivatives, phenolic acids, and hydroxycinnamoyl derivatives were higher accumulated in SC9 than SC205 at different stages (Figures 2D–G). In addition, most flavone-related DAMs were also increased in SC9 compared with SC205 from stage S1 to S3; however, this trend was not apparent during the remaining developmental stages (Figure 2H). Together, these results revealed a comprehensive change of metabolites between yellow and white cassava roots across different developmental stages.

### Developmental Effects on Metabolites in Cassava Tuberous Roots With Different Colors

**FIGURE 3:**
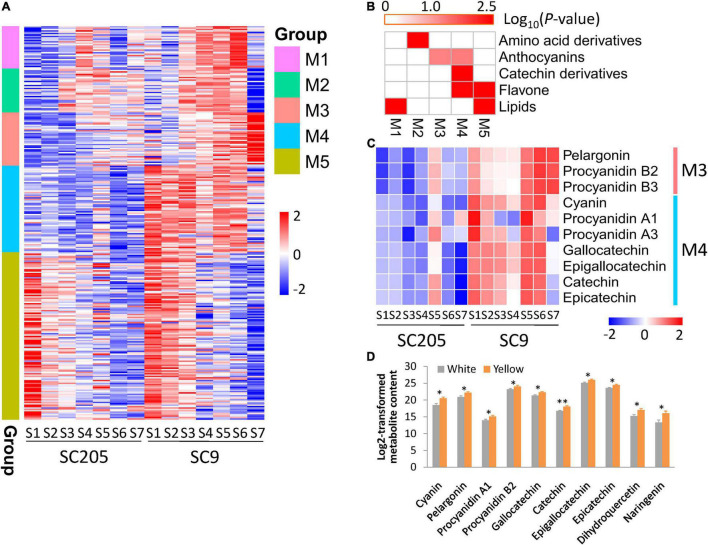
Metabolite profiles of cassava tuberous roots with different colors during seven developmental stages. **(A)** Metabolite profiles of SC205 and SC9 during tuberous root development. A total of five groups (M1-M5) were determined. **(B)** Metabolite enrichment analysis of the groups presented in panel **(A)**. **(C)** Changes of metabolites relevant to anthocyanins and catechin derivatives during tuberous root development. **(D)** DAMs identified between five white-rooted and five yellow-rooted cassava accessions at the developmental stage S7 with at least two biological replicates. The values represent the mean ± SD, and ^∗∗^ and ^∗^ represent significant differences at *P* < 0.01 and *P* < 0.05, respectively, based on the Student’s *t*-test.

A total of five groups (M1-M5) of metabolites were identified according to their profiles in SC205 and SC9 across seven different development stages (Figure 3A and Supplementary Table 2).

Metabolites from M1 were gradually accumulated from S1 to S4 and then declined until S7 in SC205, while these metabolites displayed a time-shift pattern in SC9 as they were accumulated from S1 to S6 and decreased at S7 (Figure 3A). These metabolites were significantly enriched in lipids (Figure 3B). As expected, 68% (21/31) metabolites from this group belonged to diverse forms of lysophosphatidylcholine (LPC) and lysophosphatidylethanolamine (LPE).

Metabolites from M2 were highly accumulated at late stages (S3-S6) than early stages (S1-S2) in both SC205 and SC9, while there was a sharp decrease at S7 in SC9 (Figure 3A). These metabolites were significantly enriched in amino acid derivatives (Figure 3B). A total of ten amino acids, including phenylalanine, homocysteine, methionine, tyrosine, asparagines, tryptophan, isoleucine, leucine, citrulline, and arginine, were included in this group.

Metabolites from M3 and M5 exhibited very similar patterns between SC205 and SC9 during tuberous root development, although they were highly accumulated at S4-S7 and S1-S3, respectively (Figure 3A). The metabolites from M3 were significantly enriched in anthocyanins, and anthocyanin-related metabolites such as pelargonin, procyanidin B2, and procyanidin B3 were found in this group (Figure 3C). The enriched categories of M5 were lipids and flavones (Figure 3B). A large number of metabolites relevant to monoacylglycerol (MAG, 18:1, 18:2, 18:3, and 18:4), digalactosylmonoacylglycerol (DGMG, 18:1 and 18:2), and monogalactosylmonoglyceride (MGMG, 18:2) were included in this group. In addition, flavones such as naringenin, dihydroquercetin, quercetin 3-*O*-glucoside, quercetin 3-*O*-rutinoside, kaempferol 3-*O*-galactoside, and kaempferol 3-*O*-rutinoside were also included (Supplementary Table 2).

Although no obvious trends were observed for the metabolites from M4, their levels were overall higher in SC9 than SC205. The metabolites of this group were enriched in anthocyanins, catechin derivatives, and flavones (Figure 3B). Cyanin, procyanidin A1, and procyanidin A3 relevant to anthocyanins, and gallocatechin, epigallocatechin, catechin, and epicatechin related to catechin derivatives, were found in this group (Figure 3C). Collectively, these results revealed a dynamic change of metabolites in yellow and white cassava tuberous roots during different developmental stages.

We also found that anthocyanin-related metabolites were significantly higher in SC9 than SC205 (Figure 3C). Moreover, the higher levels of anthocyanins in yellow-rooted cassava were further confirmed by metabolic comparisons between five yellow-rooted and five white-rooted cassava accessions (Figure 3D). These results suggested a significant role of anthocyanin-related metabolites in the yellow pigment formation of cassava tuberous roots.

### Transcriptomic Profiling of Cassava Tuberous Roots With Different Colors Across the Developmental Stages

The samples used for metabolic assay were subjected to RNA-seq analysis, to investigate the transcriptomic mechanisms underlying color formation of cassava tuberous roots. Principle component analysis showed that transcriptomic samples of SC9 and SC205 tuberous roots were well separated, whereas three replicates of the same sample were clustered (Figure 1C). In total, ∼1,086 million clean reads were obtained after removing the adaptors and low-quality reads, and 78.1% on average were mapped to the cassava reference genome. Low-expressed genes with FPKM < 1 across samples were discarded for further analysis.

**FIGURE 4:**
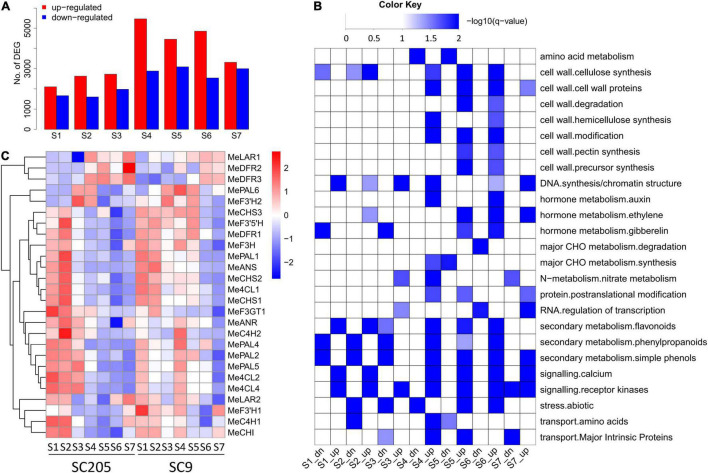
Differentially expressed genes between SC9 and SC205 during tuberous root development. **(A)** Number of up-regulated and down-regulated DEGs, which showed higher and lower expression levels in SC9 than SC205, respectively. **(B)** Functional category enrichment of the up-regulated (suffixed with “up”) and down-regulated (suffixed with “dn”) DEGs presented in panel **(A)**. **(C)** Expression profiles of anthocyanin biosynthesis genes during tuberous root development.

Differentially expressed genes (DEGs) were identified between SC9 and SC205 at each developmental stage, and functional category enrichment was subsequently analyzed for the up-regulated and down-regulated genes, respectively (Figures 4A,B). A total of 13,537 DEGs were identified (Supplementary Table 3). Overall, the number of DEGs was higher at S4-S7 than S1-S3, indicating that more genes were required to maintain the differentiation of tuberous roots between SC9 and SC205 at late developmental stages. In addition, the number of up-regulated DEGs was higher than those down-regulated at each stage, in accord with the changes of metabolites (Figures 2A, 4A). Functional enrichment found that secondary metabolism pathways (including flavonoids, phenylpropanoids, and simple phenols) were commonly enriched in the up-regulated DEGs at S1-S2 and S4-S6, which provided a strong hint to further investigate the expression of genes involved in phenylpropanoid-flavonoid (anthocyanin biosynthesis) pathways.

In total, 41 genes from fourteen key enzymes participating in anthocyanin biosynthesis were found in the cassava genome (Figure 4C and Supplementary Table 4). Fifteen of them were excluded from further analysis since they were not or low expressed during the whole developmental stages. Although similar expression patterns were observed for most of the remaining genes between SC9 and SC205, their expression levels were higher in SC9 than SC205 (especially from S3 to S6), suggesting the involvement of anthocyanin biosynthesis pathways in color formation of cassava tuberous roots.

To verify the expression levels of RNA-seq data, eleven genes involved in anthocyanin biosynthesis were examined by the qRT-PCR method in SC205. The correlation coefficients ranged from 0.86 to 0.99 between these two independent methods (Supplementary Table 1), indicating the high reliability of gene expression profiles detected by RNA-seq.

### Integrative Metabolomic and Transcriptomic Analysis for Anthocyanin Biosynthesis Pathways

**FIGURE 5:**
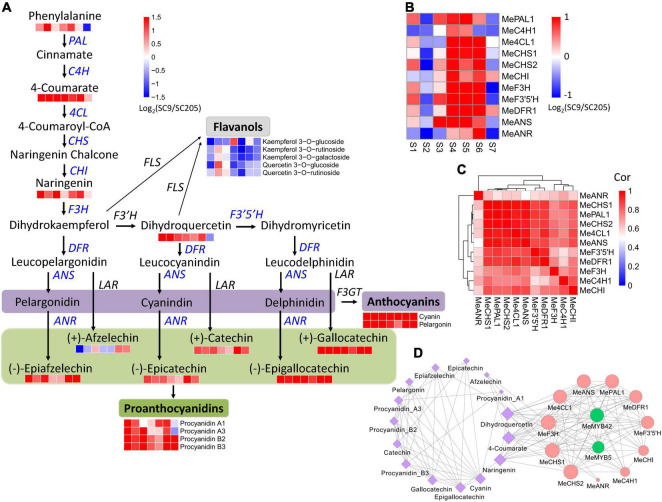
Abundance changes of metabolites and genes relevant to anthocyanin biosynthesis during tuberous root development. **(A)** Summary of anthocyanin biosynthesis pathways. Heatmaps are shown where the metabolite levels changed significantly between SC9 and SC205 during tuberous root development. Co-expressed anthocyanin biosynthesis genes are indicated in blue. **(B)** Expression fold-change of co-expressed anthocyanin biosynthesis genes between SC9 and SC205 during tuberous root development. **(C)** Expression correlation of anthocyanin biosynthesis genes shown in panel **(B)**. **(D)** The network of co-expressed genes and metabolites relevant to anthocyanin biosynthesis. The edges were shown by setting the threshold = 0.65. Node size represents its connectivity to other genes/metabolites. Metabolites are colored by purple, while anthocyanin biosynthesis genes and transcription factors are colored by red and green, respectively.

In total, 16 metabolites involved in anthocyanin biosynthesis were identified (Figure 5A), including three flavones (naringenin, dihydroquercetin, and afzelechin), six anthocyanins (cyanin, pelargonin, procyanidin A1, procyanidin A3, procyanidin B2, and procyanidin B3), five catechin derivatives (gallocatechin, epigallocatechin, catechin, epicatechin, and epiafzelechin), one amino acid (phenylalanine), and one hydroxycinnamoyl metabolite (4-coumarate). These metabolites accumulated at higher levels in SC9 than SC205 during tuberous root development. Correspondingly, eleven co-expressed DEGs (namely *MePAL1*, *MeC4H1*, *Me4CL1*, *MeCHS1*, *MeCHS2*, *MeCHI*, *MeF3H*, *MeF3’5’H*, *MeDFR1*, *MeANS*, and *MeANR*) covering the whole anthocyanin biosynthesis pathways were also identified and showed higher expression levels in SC9 than SC205 during the developmental stages (Figures 5B,C and Supplementary Table 5).

In addition, five flavanols (including kaempferol 3-*O*-glucoside, kaempferol 3-*O*-rutinoside, kaempferol 3-*O*-galactoside, quercetin 3-*O*-glucoside, and quercetin 3-*O*-rutinoside) were identified. However, they accumulated at lower levels in SC9 than SC205 during tuberous root development. Flavonol synthase (FLS) is a key enzyme involved in the conversion of dihydroflavonols (e.g., dihydrokaempferol and dihydroquercetin) to the corresponding flavonols (kaempferol and quercetin). Interestingly, in accord with the metabolic changes of the above five flavanols, we found a flavonol synthase gene (*MeFLS2*) exhibiting lower expression levels in SC9 than SC205 (Supplementary Table 4). These results suggested that *MeFLS2* was a crucial gene controlling the metabolic flows of dihydroflavonols (such as dihydrokaempferol and dihydroquercetin) toward anthocyanin biosynthesis in cassava tuberous roots.

Together, these results revealed a coordinate regulation of anthocyanin biosynthesis at the metabolomic and transcriptomic levels.

### Identification of Transcriptional Factors Modulating the Expression of Anthocyanin Biosynthesis Genes

The expression levels of genes in a co-expression network are usually regulated by the same transcriptional factors (TFs). Therefore, the eleven co-expressed anthocyanin biosynthesis genes were used as queries to perform a co-expression network analysis, to identify the crucial TFs participating in anthocyanin biosynthesis.

Two MYB members (*MeMYB5* and *MeMYB42*), whose homologs (such as *VvMYB5* and *AtMYB42*) were previously reported to be involved in anthocyanin biosynthesis regulation (29, 30), were identified to highly co-express with eight and nine anthocyanin biosynthesis genes, respectively (Figure 5D). Moreover, MYB *cis-*element was found in the 2-kb promoter region of these anthocyanin biosynthesis genes (except *MeC4H1*, Supplementary Table 5). *MeMYB5* and *MeMYB42* were also co-expressed with 4-coumarate, naringenin, and dihydroquercetin, located on the anthocyanin biosynthesis pathways (Figure 5D). These results suggested that *MeMYB5* and *MeMYB42* were the key TFs participating in the regulation of anthocyanin biosynthesis in cassava tuberous roots.

## Discussion

### Discovery of Candidate Genes and Pathways for the Coloring of Cassava Roots

It is well established that the yellow cassava roots have higher nutritional values and are more popular with consumers than white cassava roots (5, 7). Thus, the primary goal of our study is to explore key genes and pathways responsible for the color formation in yellow cassava roots. This is a crucial and fundamental step for the molecular breeding of cassava varieties with improved nutrition.

In the past decades, many progresses have been achieved concerning the mechanisms of yellow cassava roots. Carotenoid pathways were found as a major factor for yellow pigmentation, and several related genes were identified and functionally characterized (2, 9). However, the carotenoid contents were not always higher in yellow cassava roots than white cassava roots (5), indicating that other genes and pathways might be involved in the yellow pigment formation of cassava roots.

With the availability of cassava genome, the members derived from a gene family or involved in a biological pathway are identified (31). However, it is still hard to systematically determine the key players without an assistance of other omics approaches (e.g., transcriptome and metabolome), which define a biosystem at distinct molecular layers (32). Multi-omics studies have been performed to identify candidate genes and pathways controlling pigment accumulation in many plants (13, 15, 16). Similar studies were also performed in cassava in response to drought, cold, root development, and nutritional properties (6, 18, 19, 33). In this study, 355 DAMs and 13,537 DEGs were reported by comparative transcriptomic and metabolomic analyses during cassava tuberous root development between SC9 and SC205 (Supplementary Tables 2, 3). Integrated transcriptome and metabolome analyses helped in the exploration of anthocyanin metabolic pathways, since many genes and metabolites referred to the anthocyanin biosynthesis showed a coordinated change between yellow cassava roots and white cassava roots (Figure 5A). In addition, *MeFLS2* was determined as a vital gene controlling the metabolic flows of dihydroflavonols to the direction of anthocyanin biosynthesis in cassava roots (Figure 5A), in accordance with the roles of *MlFLS* in competition between anthocyanin and flavonol biosynthesis (34). By co-expression network analysis, *MeMYB5* and *MeMYB42* were identified as the key TFs to transcriptionally regulate anthocyanin biosynthesis genes in cassava (Figure 5D). These results expand our knowledge on yellow pigmentation formation in cassava tuberous roots and also suggest that multi-omics integrative analysis is a promising tool for discovering candidate genes and pathways especially with the availability of genome sequences.

### Roles of Carotenoid Synthesis Genes in the Coloring of Cassava Tuberous Roots

Carotenoid synthesis genes have been demonstrated to involve in the color formation of cassava roots (2, 5, 8). However, the full members referred to carotenoid synthesis pathways have not yet been systematically identified and their roles in color formation remain largely unknown.

**FIGURE 6:**
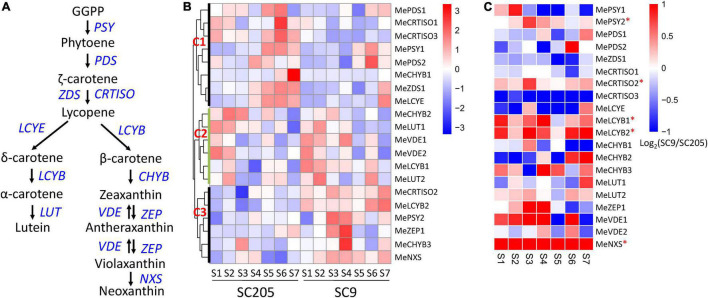
Expression changes of carotenoid biosynthesis genes during tuberous root development. **(A)** Summary of carotenoid biosynthesis pathways, in which the genes are highlighted in blue. **(B)** Expression profiles of carotenoid biosynthesis genes between SC9 and SC205 during tuberous root development. **(C)** Expression fold-change of carotenoid biosynthesis genes between SC9 and SC205 during tuberous root development. The genes suffixed with a star (^∗^) are up-regulated in SC9 than SC205 during most developmental stages and may participate in the yellow formation of cassava tuberous roots.

In this work, 23 genes were found from eleven gene families located on the carotenoid synthesis pathways in the cassava genome (Figure 6A and Supplementary Table 6). Three genes, including *MePSY3*, *MeZDS2*, and *MeZEP2*, were extremely low expressed (FPKM < 0.1) during seven stages of tuberous root development in SC205 and SC9, indicating a minimal role of these genes in cassava tuberous roots. According to their different expression patterns, the remaining 20 genes were grouped into three clusters (C1-C3, Figure 6B). The genes from cluster C1 expressed higher in SC205 than SC9, especially during S4 to S7 stages. It seems that the genes in this cluster were not relevant to the yellow formation of cassava tuberous roots; however, a previously suggested candidate gene *MeLCYE* was included in this group (8). The genes from cluster C2 exhibited similar expression patterns between SC205 and SC9, i.e., the expression levels were higher at stages S1 and S2 but lower at S3 to S7. Notably, *MeLCYB1* from this group expressed lower in SC205 than SC9 in most of the developmental stages, supporting a possible role of this gene in the color formation of cassava roots (5).

A total of six genes, including *MePSY2*, *MeCRTISO2*, *MeLCYB2*, *MeCHYB3*, *MeZEP1*, and *MeNXS*, were included in cluster C3. These genes covered most enzymes responsible for the carotenoid biosynthesis and expressed lower in SC205 than SC9 during cassava tuberous root development, with *MeLCYB2* and *MeNXS* being the most significantly changed genes (Figure 6C). These two genes exhibited significantly higher expression levels in yellow cassava roots than white cassava roots (5). In addition, their homologs have also been characterized in color formation in watermelon and Chinese kale (35, 36), indicating a similar role of these two genes in cassava roots. A key gene *MePSY2*, which was responsible for cassava roots with yellow color by provitamin A accumulation (2), was included in this group. Another carotenoid synthesis gene *MeCRTISO2*, which expressed higher in yellow cassava roots than white cassava roots (5), was also included. Moreover, positive correlations were observed between the total carotenoid content and the expression of *MeLCYB2* and *MePSY2*, respectively (5, 8). Together, these results strongly suggested that the six genes in cluster C3 might participate in the yellow pigment formation of cassava tuberous roots *via* carotenoid synthesis.

### Roles of Anthocyanin Biosynthesis Genes in Color Formation of Cassava Tuberous Roots

Anthocyanins are water-soluble pigments responsible for coloring plant flowers and fruits (10, 11). To date, anthocyanin biosynthesis genes and pathways have been demonstrated to play an important role in color formation in pepper (16), cucumber (14), asparaguses (15), jujube fruit (37, 38), cowpea (39), potato (40, 41), tea (42), longan (43), and pitaya (13). In cassava, Xiao et al. (6) found that anthocyanins and proanthocyanidins were significantly lower in white cassava roots than yellow cassava roots. Although several anthocyanin biosynthesis genes were uncovered in response to stresses and leaf and root development in cassava (18, 19, 22), the changes of anthocyanins and proanthocyanidins during cassava root development as well as the related key genes and regulatory networks remain largely unknown.

In this work, six metabolites referred to anthocyanins and proanthocyanidins were higher accumulated in yellow cassava roots than white cassava roots (Figures 3C,D). Furthermore, these metabolites exhibited distinct accumulation patterns in white and yellow cassava roots during tuberous root development (Figure 3C). To demonstrate their possible roles in color formation of cassava roots, a total of 41 anthocyanin biosynthesis genes derived from fourteen enzyme families were systematically identified throughout the cassava genome (Supplementary Table 4). Excluding fifteen low- or non-expressed genes, the majority of the remaining genes were expressed higher in SC9 than SC205. Notably, eleven DEGs covering the whole anthocyanin biosynthesis pathways were co-expressed during cassava root development (Figure 5D). *MePAL1* and *MeANS* catalyzed the first and the last steps of anthocyanin biosynthesis, respectively (44). Together with *MeF3H*, *MeCHS1*, and *MeCHS2*, these two genes ranked as the top hub genes in the co-expression network (Figure 5D), supporting that they were vital members in anthocyanin biosynthesis in cassava (45). These results also suggested that anthocyanin biosynthesis genes play a crucial role in the color formation of cassava roots *via* a coordinated expression regulation.

Anthocyanin biosynthesis genes are transcriptionally coordinated by the “MYB-bHLH-WDR (MBW)” complex that regulates their expression levels through specific *cis-*element binding in the promoter regions (46). Our co-expression network analysis revealed that *MeMYB5* and *MeMYB42* were closely related to many anthocyanin biosynthesis genes (including *MePAL1*, *MeANS*, and *MeF3H*). Moreover, MYB *cis-*element was present in the promoter region of these genes. These results suggested that *MeMYB5* and *MeMYB42* might regulate the expression of anthocyanin biosynthesis genes *via* binding to the MYB *cis-*element in cassava, in accordance with previous reporters in other species (29, 30). However, this conclusion has not been verified and deserves further investigations.

The ratio of anthocyanins and carotenoids was a major factor determining the fruit color (47). Although several crucial anthocyanin biosynthesis genes were identified to participate in color formation of cassava tuberous roots in this work, the relationships between anthocyanin and carotenoid biosynthesis genes were still not explored. Therefore, one feasible strategy for yellow-rooted cassava breeding is to examine the effect of candidate genes (especially for the TFs) individually by transgenic methods, and then to introduce multiple anthocyanin and carotenoid biosynthesis genes into a commercial cassava cultivar.

## Conclusion

In summary, the mechanisms of color formation in cassava tuberous roots were investigated by metabolomic and transcriptomic approaches during seven developmental stages. Compared with white-rooted cassava (SC205), anthocyanins, catechin derivatives, coumarin derivatives, and phenolic acids were higher accumulated in yellow-rooted cassava (SC9). Anthocyanins were particularly enriched and displayed different accumulation patterns during tuberous root development. Further analysis found that 16 metabolites participating in anthocyanin biosynthesis, as well as 11 co-expression genes covering the whole anthocyanin biosynthesis pathways, showed higher accumulation levels in SC9 than SC205 at most developmental stages, suggesting a major role of anthocyanin biosynthesis in yellow pigmentation formation of cassava tuberous roots *via* coordinate regulation. These findings expand our knowledge of anthocyanin biosynthesis on color formation in cassava tuberous roots and offer useful candidate genes for genetic breeding of yellow-rooted cassava in the future.

## Data Availability Statement

The RNA-seq datasets generated in this work were submitted to the NCBI SRA database under the accessions SRR10480882-SRR10480904 and SRR17267560-SRR17267580. The metabolomics data were deposited to the EMBL-EBI MetaboLights database with the identifier MTBLS4361.

## Author Contributions

ZD and WH designed the project and finalized the manuscript. LF, ZD, WT, JY, and YY analyzed the data. LF and ZD drafted the manuscript. All authors read and approved the final manuscript.

## Conflict of Interest

The authors declare that the research was conducted in the absence of any commercial or financial relationships that could be construed as a potential conflict of interest.

## Publisher’s Note

All claims expressed in this article are solely those of the authors and do not necessarily represent those of their affiliated organizations, or those of the publisher, the editors and the reviewers. Any product that may be evaluated in this article, or claim that may be made by its manufacturer, is not guaranteed or endorsed by the publisher.

## Footnotes

1
http://www.metware.cn/

2
http://www.bioinformatics.babraham.ac.uk/projects/fastqc/

3
http://hannonlab.cshl.edu/fastx_toolkit/index.html
